# Contrasting clinical trajectories in two ALS patients with rare FUS variants: implications for variant interpretation, penetrance, and trial readiness

**DOI:** 10.1007/s10048-026-00927-x

**Published:** 2026-10-08

**Authors:** Paula V. Gaete, Martha Peña Preciado, John Jairo Forero Díaz

**Affiliations:** 1 Neurology Department, Universidad Nacional de Colombia, Bogotá, Colombia; 2 Neurology Department, Instituto Roosevelt, Bogotá, Colombia; 3 Physical Medicine & Rehabilitation Department, Instituto Roosevelt, Bogotá, Colombia

**Keywords:** Amyotrophic lateral sclerosis, FUS, Motor neuron disease, Genetics, Case report

## Abstract

Amyotrophic lateral sclerosis (ALS) is a neurodegenerative disease that primarily affects the motor system. A minority of cases are associated with a genetic variant. Variants in *C9orf72*,* SOD1*,* TARDBP*, and *FUS* are the most common. Compared with other forms of ALS, ALS presenting with *FUS* variants is relatively aggressive. However, phenotypic heterogeneity has been reported. We describe two cases of ALS with rare *FUS* variants with contrasting clinical courses. These cases highlight the importance of the different factors that influence ALS phenotypic presentation and the challenges of variant interpretation in ALS, as outlined in the ACMG Guidelines. It is essential to recognise specific genotype-phenotype relationships and environmental factors that could alter the clinical course of the disease and affect therapeutic agent selection.

## Introduction

Amyotrophic lateral sclerosis (ALS) is a heterogeneous neurodegenerative disease that primarily affects the motor system in the cerebral cortex, brainstem and spinal cord [1]. It is a rare disease, with an estimated incidence of 0.7–3 cases per 100,000 individuals, being more common in Europe than in America or Asia [1]. ALS can be classified into familial and sporadic ALS, with approximately 10% of the patients having an affected family member. A genetic variant is found in 10–20% of sporadic ALS cases and in 50–85% of familial ALS cases [2]. More than 40 genes associated with ALS have been identified [3]. Low-frequency variants with greater effect are associated with a Mendelian inheritance pattern, incomplete penetrance, and variable expression [2]. Pathogenic genetic variants in *C9orf72*,* SOD1*,* TARDBP*, and *FUS* are the most common causes of monogenic ALS [2].

Compared with other forms of ALS, ALS presenting with *FUS* variants is relatively aggressive. However, phenotypic heterogeneity has been reported [4]. *FUS* variants are present in 3% of European familial ALS cases, 6–10% of Asian familial ALS cases and 1% of sporadic ALS cases and are associated with a juvenile onset, typically occurring before 40 years (range 11–80 years; mean age 37 years) [5]. The heterogeneity in clinical presentation depends on the specific variant, with truncating frameshift variants being associated with earlier onset and faster progression. The earlier the age of presentation, the shorter the life expectancy, with some cases even experiencing less than a year of life after the first symptoms [4]. Although cognitive changes are uncommon in patients with *FUS* variants, loss of phonemic fluency, anomia, semantic errors, perseveration, and executive dysfunction, including difficulties with planning and task shifting, have been described [6].

Here, we will discuss two patients with rare *FUS* variants and ALS with contrasting clinical presentations.

## Case 1

 Male, 39-year-old left-handed man with psoriasis and hypertension, occasional tobacco use (age 18–38), no family history of ALS or consanguinity. He presented in June 2018 with three months of progressive dysarthria and dysphagia, 6 kg weight loss over nine months, and a five-year history of right-hand action tremor. Examination showed generalized hyperreflexia, bilateral Hoffmann’s sign, persistent glabellar reflex, markedly brisk masseteric reflex, and marked atrophy of the first dorsal interosseous and the tongue with fasciculations.

Electromyography demonstrated denervation and reinnervation in bulbar, cervical, thoracic, and lumbar segments, confirming ALS by Gold Coast criteria. Brain and spinal magnetic resonance images were normal; HTLV-2 and non-treponemal tests were negative. Lumbar puncture was normal. At diagnosis, his revised-ALS Functional Rating Scale (ALSFRS-R) was 40/48, initial progression index 1.33, and FVC 87%. Functional tests performed on the patient are summarized in Table 1. It is striking that from the initial tests performed in July 2018 to the next performed in December 2018, the patient improved on those related to inferior limbs strength (6 minutes’ walk test, 30 s chair stand test, five times sit to stand test and single leg stance test), he attributed these positive changes to the start of masitinib and the more specialized physical and occupational therapy. Initially, he received vitamin C, vitamin E, and amitriptyline (as symptomatic treatment); riluzole was started but switched to edaravone after 6 months due to nausea; riluzole was later reinitiated; masitinib was started off-label but discontinued due to gastrointestinal intolerance. Symptomatic treatments included pregabalin, lidocaine patches, oral cannabis, botulinum toxin for sialorrhea, and later morphine and quetiapine (for insomnia). He received ongoing physical, occupational, respiratory, and speech therapy. This report is not designed to assess therapeutic efficacy.

**Table 1:** Functional evaluations performed across the disease progression. *m *(meters),* bpm *(beats per minute),* Kg *(kilograms)

Date	Jul-18	Dec-18	Apr-19
6 minutes’ walk test	Total distance: 526 m; Initial heart rate: 101 bpm; Final heart rate: 121 bpm Modified Borg score: 6/10	Total distance: 554 m; Initial heart rate: 76 bpm; Final heart rate: 202 bpm Modified Borg score: 5/10	Total distance: 420 m; Initial heart rate: 112 bpm; Final heart rate: 152 bpm Modified Borg score: 3/10
30-second chair stand test	14 times	16 times	14 times
Five times sit to stand test	10.17 s	7.9 s	10.3 s
Timed up and go test	8.22 s	8.63 s	10.75 s
Single-leg stance test	Right: 13.8 s; Left: 21.29 s	Right 24.41 s; Left: 31.13 s	-
Box and block test	Right hand: 72 blocks; Left hand: 76 blocks	Right hand: 61 blocks; Left hand: 61 blocks	Right hand: 47 blocks; Left hand: 53 blocks
Hand grip strength	-	Right: 11.3 Kg; Left: 22.6 Kg	Right: 8 Kg; Left: 11.3 Kg
Right manual dynamometry	-	Biceps: 10 Kg; Deltoid: 8.7 Kg; Quadriceps: 15.1 Kg; Tibialis anterior: 7.9 Kg	Biceps: 9.9 Kg; Deltoid: 4.8 Kg; Quadriceps: 12.8 Kg; Tibialis anterior: 6.0 Kg
Left manual dynamometry	-	Biceps: 11.3 Kg; Deltoid: 10.1 Kg; Quadriceps: 16.3 Kg; Tibialis anterior: 6.3 Kg	Biceps: 13.9 Kg; Deltoid: 4.7 Kg; Quadriceps: 16.8 Kg; Tibialis anterior: 5.7 Kg
Forced vital capacity	85%	80%	68%

**Fig. 1:**
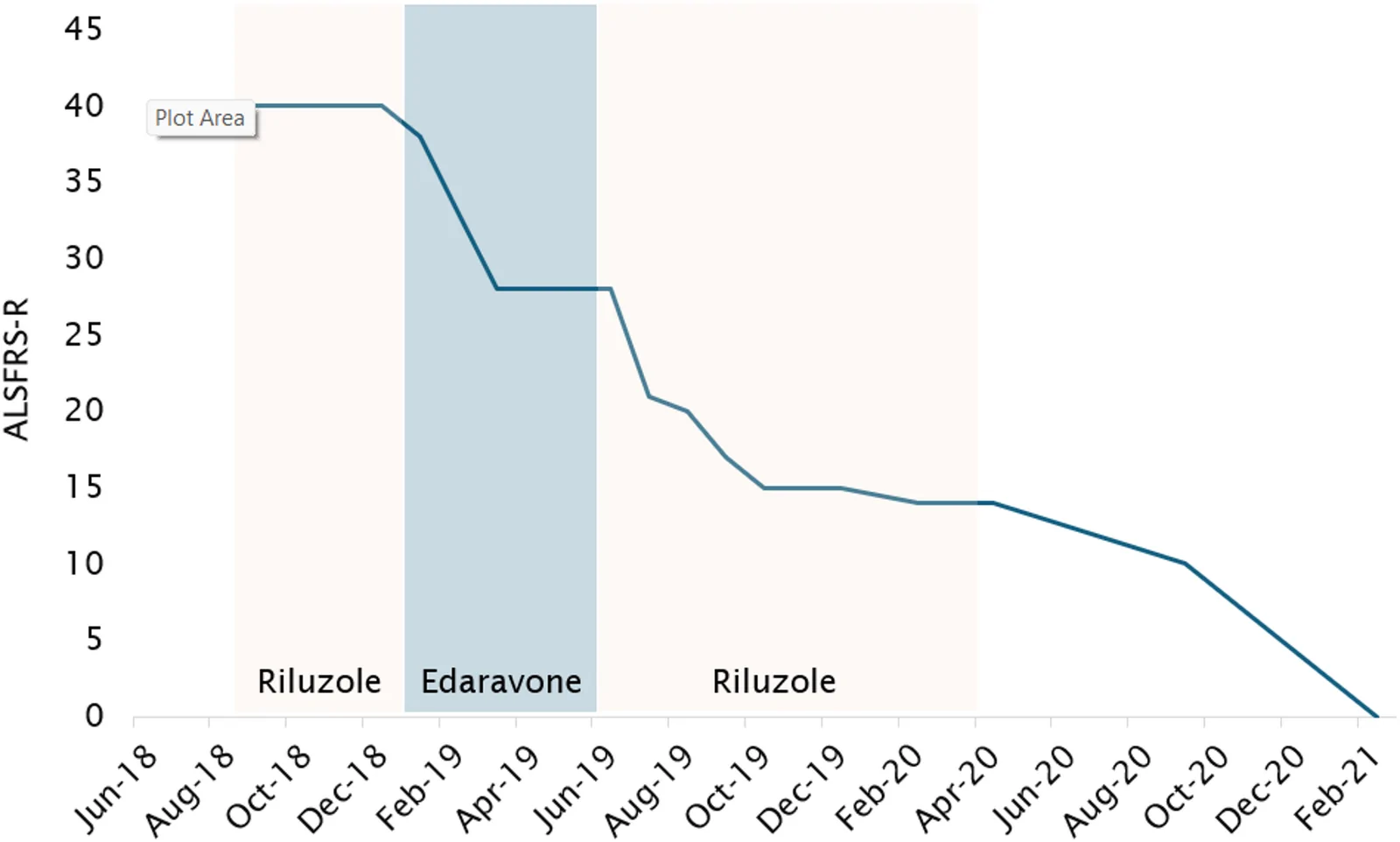
Graphic scheme of disease progression, including ALSFRS-R scores and disease modifying therapies delivered. This report is not designed to assess therapeutic efficacy

Over the following year, his disease accelerated: ALSFRS-R fell to 38/48 (Jan 2019), then 28/48 (Mar 2019) with worsening dysphagia and gait limitation; by May 2019, his FVC was 68%, and daytime BiPAP was required (progression index 2.1). By July 2019, he had severe neck muscle weakness with dropped head, marked limb weakness, left arm and cervical pain, and required a motorized wheelchair (ALSFRS-R 21/48). He declined gastrostomy and tracheostomy. By February 2020, he had severe dysarthria and dysphagia, significant dyspnea and orthopnea, generalized severe pain, sarcopenia, insomnia, and an ALSFRS-R of 14/48; morphine and quetiapine were initiated. Figure 1 shows the trajectory of the ALSFRS-R score across time.

 In the last evaluation by a neuropsychologist in April 2020, the patient reported anxiety about the future and sadness about being a burden to his family; his wife reported irritability, apathy, difficulties planning and reduced flexibility. Montreal Cognitive Assessment (MoCA) was 27/30 due to changes in attention, language fluency, and delayed recall. The Frontal Assessment Battery was normal, 18/18 points. The initial MoCA score was 30/30 in September 2018. The patient denied more detailed neuropsychological evaluations and a brain MRI (Initial brain MRI in 2018 was normal). He died at home of respiratory failure in February 2021.

Next-generation sequencing of 16 genes associated with ALS and hexanucleotide expansion analysis of *C9ORF72*, showed a short deletion in the *FUS* gene (c.521_523 + 3delGAGGTG), causing a deletion of a glycine in the G-rich region of the protein (exon 5; p.Gly174del) and changes in splicing (NM_004960.4). No other uncertain significance, likely pathogenic, or pathogenic variants were found in other genes; *C9ORF72* analysis showed fewer than 20 hexanucleotide repeats on both alleles. This variant was first described in 2009 in three patients with earlier onset ALS (age of onset: 43.3 +/- 6.4 years) [7], and then in a patient from northern England with bulbar onset ALS at age 62 associated with multiple sclerosis (since age 23) [8]. Later, another cohort study from Vancouver described this genetic variant present in four patients with sporadic ALS and in 3/678 controls, which suggests an incomplete penetrance [9]. This genetic variant has an allelic frequency of 0.0019 in GnomAD exomes. Mutation Taster predicts the mutation as disease-causing due to its low allelic frequency and potential splicing alteration, given its location at the exon-intron boundary and the expected donor-site function. In silico predictors predict a deleterious effect on protein function due to changes in the amino acid sequence (predicting the loss of the last 383 amino acids) caused by splice-site changes and an increase in nonsense-mediated decay. It is partly conserved *in Mus musculus*,* Pan troglodytes*,* Macaca mulatta*,* Drosophila melanogaster*,* Danio rerio*,* Caenorhabditis elegans and Xenopus tropicalis*. According to the large cohort and meta-analysis by Grassano et al., this case could be classified as axial ALS-*FUS* with associated bulbar symptoms [10]. The allelic frequency, ClinVar classification and the results of some in silico prediction tools for *FUS* c.521_523 + 3delGAGGTG variant are described in Table 2.

## Case 2

Female, 77 years old, without family history of ALS or consanguinity, with a history of hypertension and cardiac arrhythmia in treatment with amiodarone, losartan and hydrochlorothiazide. She reported past smoking with 30 pack years calculated (30–45 years old). She had a history of eleven years of muscular weakness in lower limbs with slow progression to upper limbs, requiring support to walk for the last 4 years. Since age 75, she had presented bulbar symptoms given by dysarthria, dysphagia, hypophonia, sialorrhea and dyspnea, with body weight loss of 10 Kg, fasciculations in the face and all limbs, cramps and cervical and lumbar pain. ALSFRS-R was calculated in 18/48 points. She was in treatment with nutritional supplementation, rehabilitation therapies (for the last five years) and riluzole (during the last month).

Physical exam at age 75 revealed inadequate cough, decreased lung expansibility with hypoventilation in the lower lobes, generalized atrophy, muscular strength of 2/5 in the MRC score in all extremities, generalized hyperreflexia, bilateral neutral plantar response, spastic dysarthria, and tongue atrophy with fasciculations. Initially, a brain tomography scan showed no structural lesions. The first electromyography, performed 1 year after symptom onset, showed large, polyphasic motor unit action potentials (MUAPs) with fasciculations in the lumbar segment. The second electromyography was performed three years later and showed the same pattern as the first, without compromise of the cervical or thoracic segments. The third and last study, performed three years later, showed polyphasic motor unit action potentials (MUAPs) associated with fasciculations and a diminished interference pattern along the cervical, thoracic, and lumbar segments. Brain and spinal magnetic resonance images were normal; HTLV-2 and non-treponemal tests were negative. Forced vital capacity was quantified at 67%, with a maximal inspiratory pressure of 65% and a maximal expiratory pressure of 40%, polysomnography showed an Apnea-Hypopnea Index of 10/hour, and a swallowing study established marked dysphagia with laryngeal penetration, but the patient rejected the gastrostomy. She began requiring oxygen supplementation at night.

Four months later, the patient started to require a wheelchair during the day and referred dropped head. Two months later, the patient started to use BiPAP with poor tolerance, for two hours a day. After two and a half years, the dysarthria progressed, and an ALSFRS-R of 17/48 points with a progression index of 0.19 was calculated. One year later, the patient began requiring support with every activity of daily living. No cognitive changes were noted during the disease progression. In June 2025, after 17 years of ALS symptoms, the patient died with severe weakness and malnutrition.

Next-generation sequencing of 32 genes associated with ALS, showed a missense variant in *FUS* gene: c.188 A > G, p.Asn63Ser (NM_004960.4). No other uncertain significance, likely pathogenic, or pathogenic variants were found in other genes; *C9ORF72* analysis showed fewer than 24 hexanucleotide repeats on both alleles. This genetic variant was first reported in 2009, in a patient with sporadic ALS from a French-Canadian population study [11]. A year later, a French study described the variant in a patient with ALS and Frontotemporal Lobar Degeneration [12]. This genetic variant has also been described in controls, suggesting reduced penetrance [13]. Mutation Taster classified this variant as non-disease-causing. Although GnomAD reports an allelic frequency of 0.000158, the amino acid position is conserved across *Mus musculus*,* Pan troglodytes*,* Macaca mulatta*,* Drosophila melanogaster*,* and Xenopus tropicalis*, and the 3-nucleotide distance from the splice site could affect splicing. According to the large cohort and meta-analysis by Grassano et al., this case could be classified as benign ALS-*FUS.* In this study, the authors showed evidence that the later onset of ALS associated with FUS is associated with a better prognosis and longer survival, as we described in the patient [10]. The allelic frequency, ClinVar classification and the results of some in silico prediction tools for *FUS* c.188 A > G, p.Asn63Ser variant are described in Table 2.

This case and others previously described with the same *FUS* variant are summarized in Table 3.

**Table 2:** Allelic frequency, ClinVar classification and in silico prediction of the impact of both *FUS* variants

Database/Tool	FUS c.521_523 + 3delGAGGTG; *p*.Gly174del	FUS c.188 A > G, *p*.Asn63Ser
gnomAD allelic frequency	0.0019	0.000158
ClinVar Classification	Benign/Likely benign	Uncertain significance/Likely benign
MutationTaster	Disease causing	Disease causing
MANE Select	Splice junction loss	Missense
BLOSUM	Not applicable	Uncertain
phyloP100	6.514	0.715
SpliceAI	Not applicable	Acceptor Loss (0.17); Donor Loss (0.06)
Pangolin scores	Not applicable	Splice Loss (0.13); Splice Gain (0.01)
AlphaMissense	Not applicable	Score: 0.0626; Points: −3; Benign
CADD	Not applicable	Score: 13.7; Points: −2; Moderate Benign
REVEL	Not applicable	Score: 0.81; Points: −2; Moderate Benign
SIFT	Not applicable	Score: 0.2; Points: −1; Supporting Benign
PolyPhen	Not applicable	Score: 0.003; Points: −2; Moderate Benign

**Table 3:** Description of case 1 and case 2 and other cases previously reported with the same *FUS* variants. *ALS *(amyotrophic lateral sclerosis),* AD *(autosomal dominant)

FUS c.521_523 + 3delGAGGTG; *p*.Gly174del						
Cite	Sample/Phenotype	Age of onset	Illness duration	Inheritance pattern	Cases	Controls
Case 1	Male, left-handed man with psoriasis, with bulbar onset ALS and right-hand action tremor	39 years	32 months	AD	Not applicable	Not applicable
[8]	Cape Verdean population study with familial ALS	43.3 +/- 6.4 years	147.0 +/- 55.2 months	AD	3/81	0/176
[9]	French Canadian population. A patient with familial ALS of bulbar onset, who had a history of multiple sclerosis since age 23. Her mother had ALS and Frontotemporal Lobar degeneration, but the patient did not present cognitive dysfunction	62 years	46 months	AD	1/159	2/293
[10]	Four Canadian patients with sporadic ALS	Not described	Not described	AD	4/168	3/678
*FUS* c.188 A > G, p.Asn63Ser						
Case 2	Female with a lumbar onset ALS	66 years	17 years	AD	Not applicable	Not applicable
[12]	French Canadian population	Not described	Not described	AD	1/200	8/190
[13]	French Study of patients with Frontotemporal Lobar degeneration and ALS	Not described	Not described	AD	1/317	0/168
[14]	Patients with sporadic ALS from Quebec and France	Not described	Not described	AD	3/454	8/450

## Discussion

Here, we described two cases of ALS associated with FUS variants. It is important to clarify that this is an association and, based on the present evidence, does not represent a causal or pathogenic relationship, given that, under ACMG criteria, neither variant is classified as pathogenic or likely pathogenic. The first case variant (*FUS* c.521_523 + 3delGAGGTG) is classified as benign, and the second case variant (FUS c.188 A > G) is classified as of uncertain significance. As established before (Tables 2 and 3), these variants have been described in ALS patients and controls, indicating reduced penetrance. However, given the contrasting clinical phenotypes, we think this information could be valuable for the management of patients with ALS and genetic variants in disease-associated genes. Considering that many patients with ALS have an association with genetic variants of uncertain significance, and functional studies are difficult to perform or not available, and most genes associated with ALS have reduced penetrance, which makes it difficult to classify the variants as pathogenic or likely pathogenic. We think this information could contribute to clarifying genotype-phenotype relationships in ALS in the future.

FUS is a DNA and RNA-binding protein highly and ubiquitously expressed that has been identified as an essential component of protein aggregates in ALS and Frontotemporal lobe degeneration [4]. FUS is predominantly localised in the nucleus and regulates RNA polymerase II activity, alternative splicing, mRNA transport and polyadenylation [14]. Transcriptomic diversity related to alternative splicing and alternative polyadenylation is particularly crucial in neurons and explains the relationship with FUS alterations and neurological diseases [14].

Many FUS genetic variants found in ALS patients are classified as of uncertain significance because of a lack of functional studies or their presence in controls [15]. Experimental studies demonstrate toxic accumulation of mutant FUS proteins, so reducing FUS expression has been proposed as a therapeutic approach for these patients. FUS antisense oligonucleotide Jacifusen targets a highly conserved sequence in intron 6 of human FUS pre-mRNA and causes degradation by RNase-H. An open-label multicentre study that included twelve participants with a *FUS* variant (pathogenic or uncertain significance) and diagnosis of ALS who received intrathecal Jacifusen showed that the therapy was safe and reduced the concentration of neurofilaments in cerebrospinal fluid, and even one patient showed objective functional recovery [5]. While case 2 would have been suitable for the study, as her FUS variant is classified as of uncertain significance, case 1 would not be, because his FUS variant is classified as benign. It is also important to note that the causal relationship between these two FUS variants and ALS development remains unclear and will require further research to be supported.

Neurofilaments are cytoskeleton proteins composed of three subunits released to the extracellular space after axonal damage. Measurements of neurofilaments in serum and cerebrospinal fluid are used to diagnose and monitor disease progression in ALS patients [16]. A meta-analysis including 122 studies and 8801 participants showed that cerebrospinal fluid neurofilaments have a sensitivity of 0.91 and a specificity of 0.9 for distinguishing ALS patients from controls. The measurement of neurofilaments in blood has a sensitivity of 0.92 and a specificity of 0.9 for differentiating ALS patients from controls. Also, higher concentrations of neurofilaments in cerebrospinal fluid or blood were associated with lower ALSFRS-R scores [17]. Another biomarker, more recently described, is peripherin; its plasma measurement discriminated between ALS patients and mimics with good accuracy at a cutoff of 1.06 ng/mL, with a sensitivity of 0.77 and a specificity of 0.767 [18]. Although no biomarkers were measured in these patients, it remains a relevant tool in the current clinical management of ALS patients and could help with diagnosis, follow-up and treatment assessment.

**Fig. 2:**
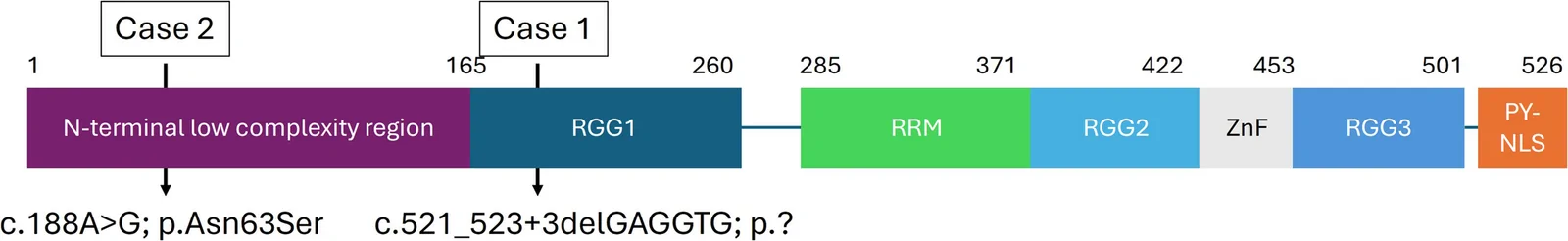
FUS domain structure and the genetic variants described in case 1 (c.521_523 + 3delGAGGTG; p.?) and case 2 (c.188 A > G; p.Asn63Ser). FUS domain structure divides the protein into an N-terminal low-complexity region (enriched in the amino acids; glutamine, glycine, serine and tyrosine), three arginine-glycine-glycine motifs (RGG1, RGG2 and RGG3, also known as rich glycine regions), an RNA recognition motif, a zinc finger domain and a nuclear localization sequence of the PY-consensus type (PY NLS) located in the C-terminal region. The first case described a patient with a genetic variant in *FUS c.521_523 + 3delGAGGTG*, causing a deletion of a glycine in RGG1 region and changes in splicing, while the second case described a patient with a missense variant in the N-terminal low complexity region of the protein. RRG: arginine-glycine-glycine motifs. RRM: RNA recognition motif. ZnF : Zinc finger domain. PY-NLS : nuclear localization sequence of the PY-consensus type

Figure 2 shows the FUS domain structure; genetic variants associated with ALS have been identified throughout the entire protein. However, the highest confidence mutations affect the RGG3 (arginine-glycine-glycine 3) or PY NLS (nuclear localization sequence of the PY-consensus type) domains. Changes in the PY-NLS domain sequence occur in 90% of patients with ALS associated with *FUS* variants and alter the protein’s nuclear transport, leading to cytoplasmic aggregation [19].

RGG motifs play a crucial role in the nuclear localisation of FUS. Arginine substitutions have been widely studied and are associated with an increased probability of cytoplasmic inclusions, while glycine residues confer greater backbone flexibility due to their smaller size and unique structure. The first case described a patient with a genetic variant in *FUS c.521_523 + 3delGAGGTG*, causing a deletion of a glycine in RGG1 region and changes in splicing. This variant could lead to persistent localization of the protein in the cytoplasm, resulting in accumulation and affecting cellular homeostasis. Mislocalization of FUS to the cytoplasm increases the probability of cytoplasmic aggregates and inhibits the translation of many proteins, upregulating non-sense-mediated decay factors ([20, 21]). The N-terminal region is essential for FUS chromatin binding, allowing the protein to regulate gene transcription and alternative splicing [22]. The second case described a patient with a missense variant in the N-terminal low-complexity region of the protein, which could affect the function of other proteins by altering alternative splicing and expression levels. It is interesting that both patients, despite their clinical presentation differences, had a clinical history of hypertension and smoking. Although it is speculative to extrapolate the evidence of epidemiological studies to the clinical course of the patients previously described, it is striking that a Mendelian randomization study published in 2014 found that increased systolic blood pressure was an independent risk factor for ALS (OR = 1.014, 95% CI 1.003–1.025, *P* = 0.015) [23], and a pooled analysis of five prospective cohorts showed a higher risk of ALS in smokers than never smokers (RR = 1.44, 95% CI 1.23–1.68; *p* < 0.0001). The risk of ALS was positively associated with pack-years smoked, smoke duration (9% increase for every 10 years of smoking; *p* = 0.006) and cigarettes smoked per day (10% increase for 10 cigarettes per day; *p* < 0.001). This is important considering that smoking exposure in the first patient is not as significant as it is in the second patient. Smoking and hypertension could be factors that modulate the penetrance of genetic variants associated with ALS, even though more evidence is required [24]. 

Additionally, in the first case, we described a young male, while in the second, a mature female. A cohort of 1.890 patients with ALS described that men with bulbar onset had shorter survival (HR 1.36, 95% CI 1.09–1.70, *p* = 0.006917), while women had a steeper functional decline [25]. The contrasting clinical trajectories observed in these two patients are likely multifactorial, and the contribution of the respective FUS variants (as opposed to age, sex, and other biological factors) cannot be determined from this case series alone. More information is required to establish causal relationships.

It is important to note that the study has limitations: we were unable to perform functional studies due to a lack of biological samples. We attempted segregation analysis, but the only family member who agreed to participate in the genetic study was the sister of case 1, who is also a carrier of the genetic variant but remains asymptomatic at 45 years old. Although the available evidence does not allow classification of these FUS variants as pathogenic or likely pathogenic under ACMG guidelines, the manuscript provides valuable information on the different ALS phenotypes associated with *FUS* variants. It is important to note that reduced penetrance has been reported even for pathogenic and likely pathogenic FUS variants [19% (95% CI, 13.0–28.4)] and for other genes associated with genetic ALS [26].

In summary, these case series highlight the various factors that influence the phenotypic presentation of ALS. Specific genotype-phenotype relationships are necessary to provide a more detailed prognosis. Classifying genetic variants for ALS poses a challenge due to their reduced penetrance and the scarcity of functional studies. Research on new biomarkers in ALS could expand the evidence for the effects of specific genetic variants on ALS clinical presentation. Moreover, environmental factors could change the clinical course of the disease and increase the probability of presenting clinical symptoms in a person with a genetic variant associated with ALS.

## Data Availability

The data that support the findings of this study are available from the corresponding author, [PVG], upon reasonable request.
